# Cross-cultural adaptation and validation of the Suffering Pictogram for Brazilian cancer patients

**DOI:** 10.1017/S147895152610176X

**Published:** 2026-02-18

**Authors:** Ana Cláudia Mesquita Garcia, Eliza Mara das Chagas Paiva, Angela Bezerra Perlamagna, Tais Rodrigues Caproni, Silvana Maria Coelho Leite Fava, Everson Meireles, Talita Prado Simão, Seng Beng Tan

**Affiliations:** 1 Interdisciplinary Center for Studies in Palliative Care, School of Nursing, Federal University of Alfenas, Alfenas, Brazil; 2 School of Medicine, Federal University of Alfenas, Alfenas, Brazil; 3 Clinical Hospital of the Faculty of Medicine, University of São Paulo, São Paulo, Brazil; 4 School of Nursing, Federal University of Alfenas, Alfenas, Brazil; 5 Health Sciences Center, Federal University of Recôncavo da Bahia, Santo Antônio de Jesus, Brazil; 6 Department of Medicine and Nursing, Federal University of Viçosa, Viçosa, Brazil; 7 Unit of Palliative Medicine, Department of Medicine, Subang Jaya Medical Center, Selangor Darul Ehsan, Malaysia

**Keywords:** Palliative care, palliative medicine, hospice care, spirituality, psychometrics

## Abstract

**Background:**

Assessing the multidimensional nature of suffering in palliative care is challenging. The Suffering Pictogram (SP) is a visual instrument developed to facilitate the communication and measurement of this experience in clinical practice.

**Objectives:**

To translate, cross-culturally adapt, and validate the SP into Brazilian Portuguese (SP-BR) for cancer patients.

**Methods:**

A sample of 222 cancer patients completed the SP-BR and the FACIT-Sp-12 scale. Psychometric properties were assessed using exploratory factor analysis (EFA), internal consistency (Cronbach’s alpha), and convergent validity (Pearson’s correlations).

**Results:**

EFA confirmed a unidimensional structure (loadings 0.40–0.73; variance explained 34.42%). Internal consistency was robust (*α* = 0.80). The SP-BR showed a moderate correlation with the FACIT-Sp-12 (*r* = −0.50, *p* ≤ 0.001).

**Conclusion:**

The SP-BR is a validated, unidimensional Brazilian Portuguese instrument suitable for holistic suffering assessment in clinical settings.

**Significance of results:**

The SP-BR is a brief tool for holistic suffering assessment, making it suitable for efficient screening in clinical and research settings, including those with limited resources.

## Introduction

Cancer is not only a physical illness but also a deeply existential challenge. Best et al. refer to cancer-related suffering as holistic suffering, understood as a multidimensional experience encompassing physical, emotional, and spiritual aspects of a person’s life (Best et al. 2015). Valid assessment of this complex experience is critical for person-centered palliative care, yet few instruments are designed to assess the diverse nonphysical dimensions of suffering in seriously ill patients (Rattner and Cait 2025), capturing its integrative nature across cultures.

The Suffering Pictogram (SP), developed by Beng et al. (2017), addresses this gap through a brief, pictogram-based tool measuring 8 domains: discomfort, worry, fear, anger, sadness, hopelessness, difficulty in acceptance, and emptiness. Pictograms, graphic symbols that convey information through illustrative representations, are designed to facilitate the comprehension of the meaning underlying the image, regardless of the individual’s literacy skills (Kolers 1969; Menon et al. 2021). The SP was primarily designed to assess experiential suffering as informed by the existing literature (Beng et al. 2017). Rather than focusing on the numerous “external” events that may occur at the end of life, the SP measures the “internal” sensory, emotional, cognitive, and spiritual experiences associated with these events (Beng et al. 2017). Its original validation demonstrated strong internal consistency (*α* = 0.84) and convergent validity with spiritual well-being (Beng et al. 2017), supporting its utility in palliative settings. The SP, designed to measure internal experiences of suffering, was created to represent the burning flames of a campfire (Beng et al. 2017).

Beng et al. (2017) highlight several advantages regarding the clinical application of the SP. It is a simple instrument to administer and score. The average duration of the test is 5 minutes, conducted through a face-to-face interview. In the original SP development study, participants did not report any difficulty answering the instrument, and 97% responded to all items (Beng et al. 2017). Moreover, the scoring procedure, based on the sum of 8 items, is easy to calculate and would not increase the workload of the healthcare team (Beng et al. 2017).

However, a critical gap persists in the Brazilian context: there is no validated instrument in Brazilian Portuguese designed to rapidly and objectively assess the diverse nonphysical dimensions of suffering in seriously ill patients. This is particularly relevant given Brazil’s distinct cultural-spiritual context, characterized by collectivist values, high religiosity, and integrated health beliefs (Moreira-Almeida et al. 2010; Lucchetti et al. 2016), which may influence how suffering is expressed and measured. Therefore, to address this need, this study aimed to translate and cross-culturally adapt the Suffering Pictogram into Brazilian Portuguese (SP-BR) and evaluate its psychometric properties in Brazilian cancer patients. Based on the original scale’s development, which identified a 2-factor structure (representing emotional-cognitive aspects of suffering), our theoretical hypothesis was that the SP-BR would replicate this bifactorial structure.

## Methods

### Study design

This is a cross-sectional, methodological study conducted in 2 stages, namely: (1) translation and cultural adaptation of the SP (Beng et al. 2017); and (2) assessment of the psychometric properties of the Brazilian version of the SP, and reported according to the STrengthening the Reporting of OBservational studies in Epidemiology (STROBE) guidelines (von Elm et al. 2007).

### Translation and cross-cultural adaptation

The translation and cross-cultural adaptation of the SP followed the rigorous, standardized procedure established by the European Organisation for Research and Treatment of Cancer (EORTC) Quality of Life Group (Kulis 2017). The process was implemented as follows: 2 independent forward translations from English into Brazilian Portuguese were performed by bilingual translators, both speakers fluent in Brazilian Portuguese and English. A reconciliation meeting was then conducted by a committee of experts composed of 5 members from the authors’ research group, all with experience in developing studies involving seriously ill patients. This committee reviewed the 2 translations against the original instrument to resolve discrepancies and produce a single synthesized Portuguese version. This version was back-translated into English by 2 other independent translators, who were blinded to the original scale. The back-translations and all translation reports were reviewed by the author of the original instrument for approval to ensure conceptual alignment. The Brazilian Portuguese version was then pilot-tested with a sample of 13 cancer patients to assess comprehensibility. During structured interviews, patients were asked to paraphrase items and identify any that were difficult, confusing, or upsetting. Their feedback was analyzed and used to refine the wording. Finally, the final version was approved by the original author of the SP.

### Setting

Participants were recruited from a general oncology service within a nonprofit organization in southern Minas Gerais, Brazil. Data collection took place in this outpatient setting with adult cancer patients.

### Population and sample

The study included adults with cancer, regardless of type, treatment, or diagnosis duration. The sample was non-probabilistic and convenience-based. For psychometric analyses, a minimum of 100 participants per assessed dimension was required, following recommendations for validity studies using exploratory factor analysis (EFA) (Damásio 2012; MacCallum et al. 1999; Pasquali 2006). Eligibility required a performance status of ≤3 (Eastern Cooperative Oncology Group (ECOG) Performance Status) and cognitive ability to respond (Oken et al. 1982). Patients with delirium or confusion, verified by the care team and researcher observation, were excluded.

### Recruitment

Participant recruitment commenced in August 2022. The study authors, who served as data collectors, received standardized training to ensure procedural consistency. Patients were approached in the common areas of the center. After explaining the study, eligibility was assessed, which included a cognitive screening based on a simple question about current news (Daté et al. 2020). Eligible patients who provided consent proceeded to complete the instruments.

### Instruments

Participant characterization: The researcher-developed questionnaire collected data on age, sex, comorbidities (e.g., hypertension, diabetes), cancer type, metastasis, treatment duration, and treatment objective (curative or palliative).

SP: The SP is an 8-item instrument developed by Beng et al. (2017) to assess multidimensional suffering from the patient’s internal perspective. It uses a Likert scale (0–4) and also includes a single-item overall suffering score (0–10). The SP demonstrated strong internal consistency (*α* = 0.84) and a strong negative correlation with Functional Assessment of Chronic Illness Therapy – Spiritual Well-Being 12 Item Scale (FACIT-Sp) (*p* = −0.63, *p* < 0.001) (Beng et al. 2017). For detailed information on the specific dimensions, factor structure, and validation, see Beng et al. (2017).

FACIT-Sp: Given the role of spiritual health in addressing holistic suffering (Beng et al. 2017), FACIT-Sp-12 was used to assess spiritual well-being. The FACIT-Sp-12 was selected to provide evidence of validity based on relations to an external variable due to its established recognition in the international literature for assessing spiritual well-being, its prior validation for the Brazilian population (Lucchetti et al. 2015), and its use in the original validation study of the SP. This provided a direct benchmark for comparison. The Brazilian Portuguese version was used with permission from the FACIT Group (Facit Group). The instrument’s total score, which is recorded on a 5-point Likert scale (higher scores indicating greater well-being), was used in correlation analyses with the SP-BR.

### Procedures

Data were collected between August 2022 and July 2023 in a private and comfortable setting. Patients completed the study questionnaires after signing informed consent. A trained researcher administered the printed instruments – the participant characterization questionnaire, SP-BR, and FACIT-Sp-12. Responses were manually recorded in Google Forms, adhering to confidentiality protocols. Completion took approximately 20 minutes.

### Data analysis

Dimensionality and evidence of validity based on the internal structure (American Educational Research Association 2014) of the SP were assessed through EFAs using the principal axis factoring (PAF) method in IBM SPSS Statistics 21. No missing values were observed for any of the instrument’s 8 items. Parallel analyses (Horn 1965), conducted using the Monte-Carlo Parallel Analysis software (Watkins, 2000), served as the criterion for determining the number of factors to extract from the data matrix. The Kaiser–Meyer–Olkin (KMO) index was used to verify sample adequacy for factor analysis – criterion KMO ≥ 0.70 (Damásio 2012). The criteria adopted for item retention in the factor were as follows: factor loading and item-total correlation ≥ 0.32; theoretical plausibility of item grouping (Hair et al., 2010; Rios and Wells 2014). Internal consistency was assessed in SPSS 21 using Cronbach’s alpha coefficient, with values above 0.70 considered acceptable for exploratory studies (Hair et al., 2010).

Validity evidence based on relationships with external variables was examined through hypothesis-driven Pearson correlations, performed in SPSS 21. Following Akoglu (2018), correlation coefficients were interpreted as: 0.1–0.3 (weak), 0.4–0.6 (moderate), and 0.7–0.9 (strong). We tested 2 pre-specified hypotheses: (1) SP-BR scores would show a strong positive correlation (*r* ≥ 0.70) with the overall suffering score (0–10 scale), reflecting concurrent validity; (2) SP-BR scores would show a strong negative correlation (*r* ≤ −0.70) with FACIT-Sp-12 scores, reflecting divergent validity (spiritual well-being being a theoretically inverse construct to suffering). Statistical significance was set at *p* < 0.05. Descriptive statistics summarized the instrument scores. No missing data were observed for any items of the SP-BR or the FACIT-Sp-12, as all instruments were completed in full by the 222 participants.

## Results

### Sample characterization

A total of 222 patients participated in the quantitative phase of the study, completing the SP-BR and FACIT-Sp-12. The mean age of participants was 59.84 years (range: 22–90, SD = 12.7), and the mean duration of cancer treatment was approximately 42 months (range: 2–144, SD = 31.7). Additional characterization data are presented in Table 1. While the full sample (*n* = 222) was used for all psychometric analyses, sociodemographic and clinical characterization data were available for a subset of 204 participants, as detailed in Table 1 (see footnote).

**Table 1.** Sample characterization (*n* = 204)^a^

Variables		*n* (%)
Gender	Female	148 (72.5)
	Male	56 (27.5)
Type of cancer	Breast	81 (39.7)
	Prostate	21 (10.3)
	Gastrointestinal	32 (15.7)
	Head and neck	13 (6.4)
	Female reproductive system	9 (4.4)
	Other	48 (23.5)
Presence of metastasis	Yes	51 (25)
	No	119 (58.3)
Main treatment purpose	Curative	165 (80.9)
	Palliative	39 (19.1)

a Due to an operational problem at the start of data collection, it was not possible to obtain characterization data for 18 participants.

### Translation and cultural adaptation

The back-translated versions of the SP-BR were sent to the first author of the original scale publication for approval (Beng et al. 2017), ensuring conceptual equivalence (T. S. Beng, personal communication, November 18, 2022). The complete process of translation and cultural adaptation and the final version of the SP-BR are provided as supplementary material.

### Psychometric properties – dimensionality analysis of the Brazilian version of the Suffering Pictogram

**Figure 1.**
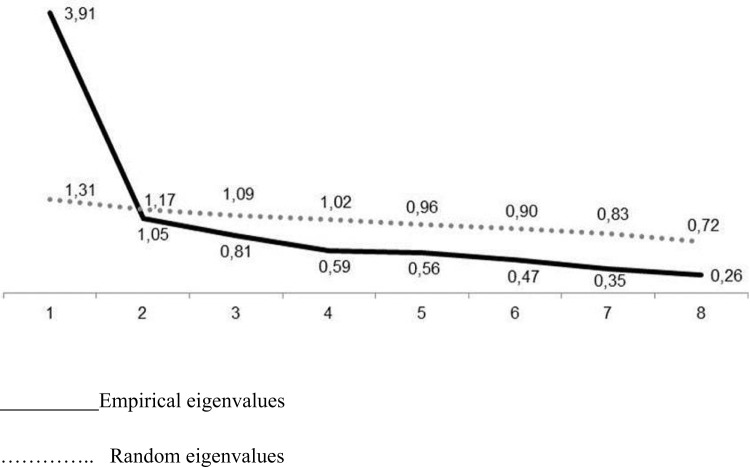
Parallel analysis sedimentation graph for determining the number of factors to extract from the data matrix.

The results of parallel analyses indicated that it was appropriate to extract just one factor. From the first factor onward, the random eigenvalues exceed the empirical ones, indicating no plausibility for more than one factor (Figure 1).

The dimensionality analysis yielded a KMO measure of sampling adequacy of 0.83, indicating sufficient linear intercorrelations among the variables for conducting EFA (Hair et al. 2009). The EFA results, obtained using the PAF method, are presented in Table 2. The items aligned appropriately with a unidimensional structure, all with loadings ≥0.40 (Table 2). The internal consistency of this grouping, assessed using Cronbach’s alpha, was satisfactory (*α* = 0.80).

**Table 2.** Results of the exploratory factor analysis of the SP-BR (*n* = 222)

Items	Factor loadings	Item-total correlation
Sadness	0.73	0.39
Emptiness	0.73	0.60
Worry	0.66	0.46
Difficulty in acceptance	0.63	0.34
Fear	0.52	0.64
Hopelessness	0.51	0.45
Discomfort	0.43	0.55
Anger	0.40	0.63
Number of items		08
Total variance explained (%)		34.42%
Cronbach’s alpha (*α*)		0.80

Extraction method: principal axis factoring. Converged in 5 interactions.

The correlation between the SP-BR score (mean = 1.30, standard deviation = 0.79, median = 1.25) and the overall suffering rating assigned by participants indicated a moderate, positive, and highly significant relationship, demonstrating high convergence (*r* = 0.62, *p* ≤ 0.001). The total scores of the FACIT-Sp-12 items (with items 4 and 8 reversed; *α* = 0.85) were calculated and correlated with the SP scores. The results indicate a moderate, negative, and highly significant correlation between the total scores of the FACIT-Sp-12 and the SP-BR (*r* = −0.50, *p* ≤ 0.001), suggesting that higher levels of suffering, as measured by the SP-BR, are associated with lower levels of spiritual well-being, as measured by the FACIT-Sp-12 (Table 3). The participants reported moderate overall suffering (mean [*M*] = 4.44, standard deviation [SD] = 3.22) with significant variability (0–10). The SP-BR items with the highest scores were *Worry* (*M* = 2.26, SD = 1.29) and *Discomfort* (*M* = 1.74, SD = 1.23), while *Hopelessness* (*M* = 0.53, SD = 0.97) and *Anger* (*M* = 0.69, SD = 1.07) had the lowest scores.

**Table 3.** Correlations between the SP-BR and FACIT-Sp-12 (*n* = 222)

SP-BR	FACIT-Sp-12
Discomfort	−0.22**
Worry	−0.32**
Fear	−0.12
Anger	−0.31**
Sadness	−0.29**
Hopelessness	−0.48**
Difficulty in acceptance	−0.51**
Emptiness	−0.40**
Factor score – SP-BR	−0.50**

** *p* ≤ 0.001.

## Discussion

This study successfully translated, cross-culturally adapted, and validated the SP-BR for use in palliative care settings. The SP-BR demonstrated strong psychometric properties, including a stable unidimensional structure, clinically meaningful convergent validity with both overall suffering (*r* = 0.62) and spiritual well-being measures (*r* = −0.50), and robust internal consistency (*α* = 0.80). These findings affirm the tool’s reliability and validity for capturing the multidimensional nature of suffering in Brazilian cancer patients, while revealing culturally distinct conceptualizations, particularly the holistic integration of physical sensations (discomfort) into the suffering construct, contrasting with the original scale’s structure (Beng et al. 2017).

Our theoretical hypothesis, based on the original scale’s development, was that the SP-BR would replicate a bifactorial structure. However, the EFA consistently supported a unidimensional model, which represents the most significant structural divergence from the original instrument. While the original EFA by Beng et al. (2017), using PAF with Promax rotation, suggested 2 factors (emotions: *α* = 0.72, worry, fear, sadness; and cognitions: *α* = 0.79; hopelessness, emptiness, difficulty in acceptance, anger), excluding “discomfort” (factor loading < 0.50), our analysis did not support this distinction, and all 8 items, including “discomfort,” were retained with significant loadings. Although methodological factors, such as sample characteristics or assessment context, could contribute to this discrepancy, the finding invites a cultural interpretation. One plausible explanation is that within Brazil’s collectivist context, suffering is conceptualized in a more holistic and integrated manner, without clear boundaries between its emotional, cognitive, and spiritual dimensions, a perspective supported by theoretical frameworks on culture and suffering (Krikorian and Limonero 2012). Therefore, we interpret the unidimensionality not as a statistical anomaly but as a potential reflection of a culturally specific phenomenology of suffering, though this requires further investigation through mixed-methods and cross-cultural comparative studies.

Regarding evidence of validity based on external variables, our results indicate that correlations with the general suffering score (*r* = 0.62, *p* ≤ 0.001) and FACIT-Sp-12 (*r* = −0.50, *p* ≤ 0.001) further confirm concurrent validity. Although we hypothesized strong correlations (*r* ≥ 0.70) based on Akoglu (2018), the observed moderate correlations (SP-BR vs. overall suffering: *r* = 0.62, SP-BR vs. FACIT-Sp-12: *r* = −0.50) remain clinically meaningful. The convergence with overall suffering (*r* = 0.62) exceeds the threshold for concurrent validity (*r* ≥ 0.50) (Portney and Watkins 2007; Wood et al. 2025), supporting SP-BR’s utility. The moderate negative correlation with FACIT-Sp-12, as in the Pictogram development study (-0.562, p < 0.001) (Beng et al., 2017) is consistent with the literature regarding the use of spiritual resources in coping with suffering and illness (Tognacci 2021; Vitorino et al. 2023).

Our findings align with and reinforce the conclusions of the systematic review by Gutiérrez-Sánchez et al. (2020), which identified the SP as the instrument with the best methodological quality and quality of evidence for assessing suffering in palliative care. Our adaptation for Brazilian Portuguese confirmed a robust unidimensional structure and good internal consistency (*α* = 0.80), mirroring the strong psychometric properties (*α* = 0.836) reported in the Malaysian population (Beng et al. 2017). This consistency across cultural adaptations underscores the instrument’s conceptual stability as a measure of holistic suffering. This near-identical Cronbach’s alpha coefficient indicates that the SP-BR maintains the measurement precision of its source instrument despite transcultural adaptation. Both values exceed the threshold of 0.70 recommended for group-level comparisons in clinical research (Hair et al. 2009), confirming the tool’s statistical reliability for assessing suffering in Brazilian populations. This consistency is particularly notable given the retention of all 8 items – including “discomfort,” which showed lower factor loading (0.43) but contributed to the scale’s conceptual comprehensiveness in capturing physical-spiritual distress integration within the Brazilian context.

The brevity (8 items) and simplicity of the SP-BR make it feasible for rapid assessment in resource-limited settings and allow its immediate integration into clinical workflows. In high-volume services, such as those in Brazil’s Unified Health System (Sistema Único de Saúde), the instrument can be used as an initial screening tool to identify critical domains of suffering (e.g., “Worry” and “Discomfort”). High SP-BR scores combined with low spiritual well-being scores, as observed in our study, indicate the need for spiritual interventions, justifying referrals to multidisciplinary teams (e.g., psychologists, chaplains). Clinicians can use the SP-BR not only for assessment but also to monitor responses to palliative interventions (e.g., after analgesia adjustment or spiritual counseling). Its unidimensional structure simplifies interpretation, facilitating communication among non-specialist professionals, which is particularly valuable in regions with a shortage of palliative care specialists.

According to The Lancet Oncology Commission (Rodin et al. 2025), the psychological and emotional distress in patients with serious illnesses such as cancer is often overlooked by healthcare systems. Simple strategies like implementing screening tools can identify this nonphysical suffering and guide interventions to mitigate it (Rodin et al. 2025). In this sense, our findings support the incorporation of the SP-BR into clinical practice, in line with the Brazilian National Palliative Care Policy (Política Nacional de Cuidados Paliativos – PNCP) (Brasil. Ministério da Saúde 2024). The SP-BR offers a practical solution for implementing Article 8 of the PNCP, which states that “In the context of palliative care, care teams and professionals within the RAS care network must conduct comprehensive patient assessments to promote relief of pain and other symptoms, considering physical, psychological, emotional, spiritual, and social needs.” Validated as a multidimensional instrument, the SP-BR facilitates the direct operationalization of this guideline by enabling comprehensive mapping of holistic suffering domains (sensory, emotional, cognitive, spiritual) in a short period of time and by identifying critical imbalances between dimensions (e.g., high physical “Discomfort” + low spiritual well-being) that require targeted interventions. Furthermore, the instrument generates standardized data both for patient suffering assessment and for evaluating outcomes following clinical interventions.

This study presents notable strengths. First, it rigorously followed international guidelines for cross-cultural adaptation (EORTC Translation Procedure), ensuring linguistic and conceptual equivalence of the SP-BR. The approval of the back-translated version by the original scale developer further strengthens content validity. Psychometric evaluation adhered to contemporary standards for factor analysis and validity testing, with a sample size exceeding recommendations for EFA (≥100 participants) (MacCallum et al. 1999; Pasquali 2006; Damásio 2012). The inclusion of patients across cancer types and treatment stages (curative and palliative) enhances clinical representativeness. The unidimensional structure of the SP-BR (*α* = 0.80) suggests practical utility in time-constrained clinical settings.

Despite the robust psychometric properties demonstrated in this study, some methodological limitations warrant a critical interpretation of the findings. The reliance on a convenience sample from a single, nonprofit center limits the demographic and clinical variability of our participants, which may affect the stability of the unidimensional factor structure and compromise the generalizability of the SP-BR to broader Brazilian healthcare contexts, such as public hospitals or rural areas. Furthermore, the cross-sectional design, while suitable for initial validation, precludes the assessment of 2 fundamental psychometric properties: test–retest reliability, which is essential for establishing the instrument’s stability over time, and responsiveness, which is critical for evaluating its sensitivity to clinical change in palliative care settings. The absence of these analyses means the longitudinal utility of the SP-BR remains unverified. Finally, future studies should employ confirmatory factor analysis (CFA) on an independent sample to provide a rigorous test of the unidimensional model suggested by our EFA and to establish measurement invariance across different patient subgroups.

As the first validated Brazilian Portuguese instrument for holistic suffering assessment, the SP-BR provides a foundation and a validated starting point for future research and adaptation across the Lusophone world. Its successful adaptation reinforces the need for investment in cross-cultural research on brief instruments for resource-limited contexts. For future research, we recommend conducting CFAs and test–retest reliability studies to further strengthen the psychometric evidence.

## Conclusion

This study successfully adapted and validated the SP-BR, establishing it as a psychometrically sound instrument to assess multidimensional suffering in patients with cancer. Its demonstrated unidimensional structure and good internal consistency support its cultural appropriateness for this population. The SP-BR’s brevity and intuitive format make it a feasible tool for rapid clinical screening, offering a practical means to integrate holistic suffering assessment into routine palliative care in Brazil.

## Supporting information

10.1017/S147895152610176X.sm001 Garcia et al. supplementary material 1 Garcia et al. supplementary material

10.1017/S147895152610176X.sm002 Garcia et al. supplementary material 2 Garcia et al. supplementary material

## Supplementary material

The supplementary material for this article can be found at https://doi.org/10.1017/S147895152610176X.
