# Supplementary material for: Cross-cultural adaptation and validation of the Suffering Pictogram for Brazilian cancer patients
Source: Palliat Support Care. 2026 Feb 18;24:e60. doi: 10.1017/S147895152610176X (PMC13166607; doi:10.1017/S147895152610176X)
Supplement: Garcia et al. supplementary material 1 — Garcia et al. supplementary material [file S147895152610176Xsup001.pdf]

Permission Statement for Use of the Suffering Pictogram

I, Dr Tan Seng Beng, am the original creator and full copyright holder of the Suffering Pictogram, first published in 2017 in *Journal of Palliative Medicine*. I have not transferred copyright or granted exclusive rights to any publisher or third party.

I hereby grant full permission to the authors of the manuscript entitled **“Brazilian Validation of the Suffering Pictogram”** to translate, culturally adapt, validate, reproduce, and publish the Suffering Pictogram in both print and electronic formats as part of this research study. This permission extends to any subsequent reproductions, derivative works, and scholarly or clinical uses arising from this study, provided that proper attribution is given to the original source.

Signed,

Dr Tan Seng Beng
16th August 2025
