# Supplementary material for: Cross-cultural adaptation and validation of the Suffering Pictogram for Brazilian cancer patients
Source: Palliat Support Care. 2026 Feb 18;24:e60. doi: 10.1017/S147895152610176X (PMC13166607; doi:10.1017/S147895152610176X)
Supplement: Garcia et al. supplementary material 2 — Garcia et al. supplementary material [file S147895152610176Xsup002.pdf]

Translation and Cultural Adaptation – *The Suffering Pictogram*

Item	Original version	Translation 1 (English > Brazilian Portuguese)	Translation 2 (English > Brazilian Portuguese)	Reconciliation	Backtranslation 1 (Brazilian Portuguese > English)	Backtranslation 2 (Brazilian Portuguese > English)	Pilot testing	After pilot testing
Title	The Suffering Pictogram	O Pictograma do Sofrimento	Pictograma do Sofrimento	Pictograma do Sofrimento	Suffering Pictogram	Pictogram of Suffering	Pictograma do Sofrimento	Pictograma do Sofrimento
Explanations	Please rate your current experience of suffering by shading the pictogram.<br><br>0 = None (Shade area 0)<br>1 = A little bit (Shade area 0 and 1)<br>2 = Somewhat (Shade area 0, 1 and 2)<br>3 = Quite a bit (Shade area 0, 1, 2 and 3)<br>4 = A lot (Shade area 0, 1, 2, 3 and 4)<br><br>Please write your overall suffering score from 0-10 at the centre of the pictogram.<br>(0 = None, 10 = Worst possible suffering)	Por favor, avalie a sua experiência atual de sofrimento pintando o pictograma.<br><br>0 = Nenhum (Pinte a área 0)<br>1 = Quase nada (Pinte as áreas 0 e 1)<br>2 = Um pouco (Pinte as áreas 0, 1 e 2)<br>3 = Bastante (Pinte as áreas 0, 1, 2 e 3)<br>4 = Muito (Pinte as áreas 0, 1, 2, 3 e 4)<br><br>Por favor, dê uma nota geral para o sofrimento numa escala de 0 a 10 no centro do pictograma.<br>(0 = Nenhum, 10 = Pior sofrimento possível)	Avalie por favor sua atual experiência de sofrimento preenchendo o pictograma<br><br>0 = Nenhum<br>1 = Muito pouco<br>2 = Considerável<br>3 = Muito sofrimento<br>4 = Sofrimento ao extremo<br><br>Por favor, dê uma nota geral para o sofrimento numa escala de 0 a 10 no centro do pictograma.<br>(0 = Nenhum, 10 = Pior sofrimento possível)	Por favor, avalie a sua experiência atual de sofrimento preenchendo o pictograma.<br><br>0 = Nenhum (Preencher a área 0)<br>1 = Quase nada (Preencher as áreas 0 e 1)<br>2 = Um pouco (Preencher as áreas 0, 1 e 2)<br>3 = Muito (Preencher as áreas 0, 1, 2 e 3)<br>4 = Muitíssimo (Preencher as áreas 0, 1, 2, 3 e 4)<br><br>Por favor, dê uma nota geral para o seu sofrimento numa escala de 0 a 10 no centro do pictograma.<br>(0 = Nenhum, 10 = Pior sofrimento possível)	Tell us what your actual level of suffering by filling this pictogram<br><br>0 none (fill the space 0)<br>1 - almost nothing (fill the spaces 0 and 1)<br>2 - a little bit (fill the spaces 0, 1 and 2)<br>3 - a lot (fill the spaces 0, 1, 2 and 3)<br>4 - very much (fill the spaces 0, 1, 2, 3 and 4)<br><br>On a scale of 0 to 10, give us your level of suffering in the midle of the pictogram<br>(0 none, 10 worst suffering ever)	Please, evaluate your actual experience of suffering filling in pictrogram.<br><br>0 = None (Fill the 0 area)<br>1 = Almost nothing (Fill the 0 and 1 areas)<br>2 = A little (Fill the 0, 1 and 2 areas)<br>3 = A lot (Fill the 0, 1, 2 and 3)<br>4 = Very much (Fill the 0, 1, 2, 3 and 4 areas)<br><br>Please, give a general note for your suffering in a scale of 0 to 10 in the center of the pictogram. (0= None, 10= Worst suffer possible).	Por favor, avalie a sua experiência atual de sofrimento preenchendo o pictograma.<br><br>0 = Nenhum (Preencher a área 0)<br>1 = Quase nada (Preencher as áreas 0 e 1)<br>2 = Um pouco (Preencher as áreas 0, 1 e 2)<br>3 = Muito (Preencher as áreas 0, 1, 2 e 3)<br>4 = Muitíssimo (Preencher as áreas 0, 1, 2, 3 e 4)	Por favor, avalie a sua experiência atual de sofrimento preenchendo o pictograma.<br><br>0 = Nenhum (Preencher a área 0)<br>1 = Quase nada (Preencher as áreas 0 e 1)<br>2 = Um pouco (Preencher as áreas 0, 1 e 2)<br>3 = Muito (Preencher as áreas 0, 1, 2 e 3)<br>4 = Muitíssimo (Preencher as áreas 0, 1, 2, 3 e 4)
1	Discomfort	Desconforto	Desconforto	Desconforto	Discomfort	Discomfort	Desconforto	Desconforto

2	Worry	Preocupação	Preocupação	Preocupação	Preoccupation	Preoccupation	Preocupação	Preocupação
3	Fear	Medo	Medo	Medo	Fear	Fear	Medo	Medo
4	Anger	Raiva	Raiva	Raiva	Anger	Anger	Raiva	Raiva
5	Sadness	Tristeza	Tristeza	Tristeza	Sadness	Sadness	Tristeza	Tristeza
6	Hopelessness	Desesperança	Falta de esperança	Falta de esperança	Lack of hope	Hopelessness	Falta de esperança	Falta de esperança
7	Difficulty in acceptance	Dificuldade em aceitar	Dificuldade de aceitação	Dificuldade em aceitar	Having a hard time accepting things	Difficulty in accept	Dificuldade em aceitar	Dificuldade em aceitar
8	Emptiness	Sentimento de estar vazio	Sentimento de vazio emocional	Sentimento de vazio emocional	Feeling of emotional emptiness	A felling of emotional emptiness	Sentimento de vazio emocional	Sentimento de vazio emocional
---	---	---	---	---	---	---	---	Por favor, assinale abaixo uma nota geral para o seu sofrimento numa escala de 0 a 10 (0 = Nenhum, 10 = Pior sofrimento possível):

Pictograma do Sofrimento

Por favor, avalie a sua experiência atual de sofrimento preenchendo o pictograma.

0 = Nenhum (Preencher a área 0)
1 = Quase nada (Preencher as áreas 0 e 1)
2 = Um pouco (Preencher as áreas 0, 1 e 2)
3 = Muito (Preencher as áreas 0, 1, 2 e 3)
4 = Muitíssimo (Preencher as áreas 0, 1, 2, 3 e 4)

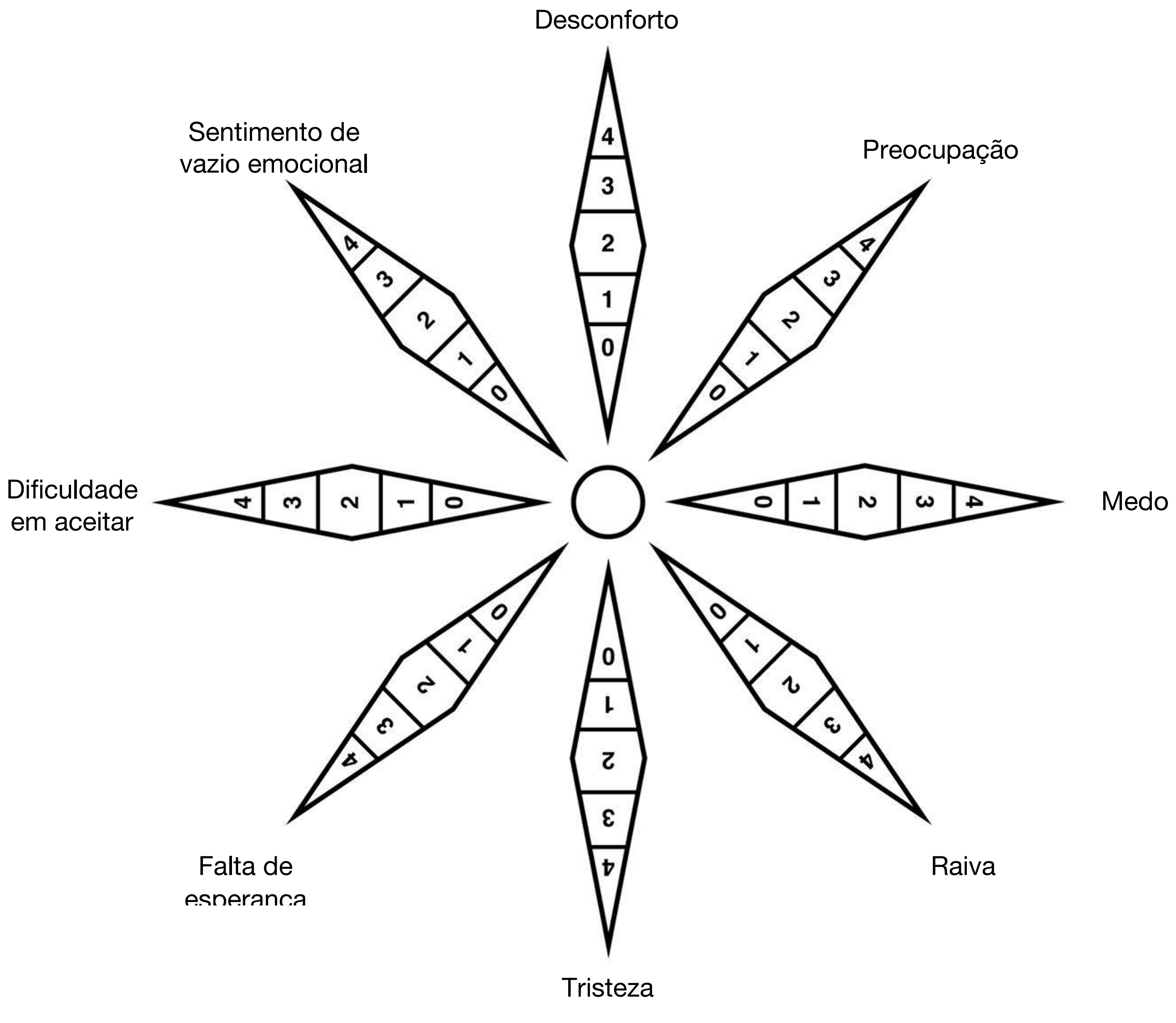

Por favor, assinale abaixo uma nota geral para o seu sofrimento numa escala de 0 a 10 (0 = Nenhum, 10 = Pior sofrimento possível):

0	1	2	3	4	5	6	7	8	9	10
